# Identification of human exposure to airborne micro-plastics and its potential effects utilizing bronchoalveolar lavage (BAL) fluid

**DOI:** 10.6026/973206300222074

**Published:** 2026-04-30

**Authors:** Tanya Jain, Meenal Jain, Rajat Nayak, Talha Saad, Akash Wamanrao Karale, Vrashabhan Ahirwar, Shailendra Patel

**Affiliations:** 1 Department of Anaesthesiology, R. D. Gardi Medical College, Ujjain, Madhya Pradesh, India; 2 Department of Anaesthesiology, Bundelkhand Government Medical College Sagar, Madhya Pradesh, India; 3 Department of Emergency Medicine, Bundelkhand Government Medical College Sagar, Madhya Pradesh, India; 4 Department of Respiratory Medicine, Bundelkhand Medical College Sagar, Madhya Pradesh, India; 5 Department of Surgery, Bundelkhand Medical College, Sagar, Madhya Pradesh, India; 6 Department of Radiology, Bundelkhand Government Medical College, Sagar, Madhya Pradesh, India; 7 Department of Forensic Medicine and Toxicology, Bundelkhand Medical College, Sagar, Madhya Pradesh, India

**Keywords:** Airborne micro-plastics, respiratory tract, bronchoalveolar lavage (BAL) fluid, environmental exposure

## Abstract

Airborne micro-plastics can be inhaled and remain in the lower respiratory tract due to their small size and tendency to remain in the air in turn posing a lethal threat to human life. Therefore, it is of interest to detect airborne micro-plastics in bronchoalveolar lavage (BAL) fluid and to assess their association with lung function, airway inflammatory cell profile and respiratory tract symptoms in adult subjects with indiscernible symptoms. BAL fluid samples of ninety-two adults were collected using strict contamination-controlled protocols and filtered via filters with a 0.45 µm membrane. Pulmonary function was assessed using spirometry in accordance with ATS guidelines. 69 (75%) respondents reported the presence of airborne micro-plastics in BAL fluid. Respondents with micro-plastics in BAL exhibited lower FEV_1_, FVC and FEV_1_/FVC ratios compared with micro-plastic-negative participants (p < 0.001). Data helps to comprehend the ill effects of micro-plastics on respiratory health.

## Background:

The tiny particles less than 5 mm are considered micro-plastics, which are raising concerns worldwide. The literature has shown that many studies have been conducted on marine and food contamination, but the focus has now shifted towards environmental exposures. Airborne micro-plastics have been poorly acknowledged. Urban air has been shown to contain large quantities of synthetic fibres and plastic fragments that are easily inhaled, raising concern about their potential deposition within the human respiratory tract and their long-term health implications. Airborne micro-plastics have been detected both inside and outside the air, specifically in heavily populated cities, with synthetic textiles, packaging materials and vehicle emissions acting as major sources, as discovered in recent research [1]. In their research, Rybalchenko *et al.* reported that, due to the small size of micro-plastics and their aerodynamic properties, they can bypass airway defence mechanisms and penetrate bronchioles and alveolar areas of the lung [2, 3]. However, existing literature remains fragmented. Various researchers have discovered micro- plastics in BAL fluid but have not established an association with lung function or the severity of symptoms [4] limited clinical studies evaluating micro-plastic burden in BAL fluid in symptomatic adults. Lack of association between exposure to micro- plastics and parameters affects pulmonary function. Literature evidence shows sparse data linking airway inflammatory cell profiles with micro-plastic presence [5, 6]. BAL fluid provides a unique biological matrix for direct assessment of lower-airway deposition and inflammatory responses. Therefore, it is of interest to evaluate spirometry assessment and inflammatory profiling, with the identification of micro-plastics, was pursued to provide detailed evidence on the health effects of airborne micro-plastics.

## Materials and Methods:

## Study design and sample:

This is a cross-sectional research analysis conducted with 92 respondents over 1 year, from January 2023 to December 2023. The following were the inclusions and exclusions of the present research analysis.

## Inclusion criteria:

[1] Age between 18 and 65 years

[2] Patients undergoing bronchoscopy for chronic cough, dyspnoea, non-resolving pneumonia or unexplained pulmonary infiltrates

[3] No known occupational exposure to industrial plastics, textile fibres or chemical dust

[4] Willingness to provide written informed consent

## Exclusion criteria:

[1] Active pulmonary infections (tuberculosis, pneumonia, COVID-19)

[2] Known malignancy

[3] History of smoking in the past 5 years

[4] Recent hospitalization (<1 month)

[5] Patients on long-term corticosteroids or immunosuppressive therapy

## Sample size:

Sample size was calculated using a prevalence-based formula: (see PDF)

Where:

[1] Z=1.96 (for 95% confidence level)

[2] p=Expected prevalence of BAL micro-plastic detection = 70% (0.70)

[3] q=1-p=0.30

[4] d= Absolute precision = 10% (0.10)

Adding 15% for non-response and sample loss:

n=80.64+12.1≈92

Thus, the final sample size = 92 participants.

## Procedure:

Bronchoscopy was performed using standard sterile procedures. During the approach, normal sterile saline was administered into the desired bronchial region to perform BAL, which was then carefully drained. BAL fluid samples were collected in glass containers and transported immediately for laboratory analysis. Before analysis, the samples were filtered using glass-based filtration systems. Organic material was removed using established digestion protocols to facilitate accurate visualization and identification of micro-plastic particles. Filtered specimens were subsequently inspected for possible tiny plastic particles using polarized light microscopy. To enhance particulate recognition, luminous staining techniques were employed. Spectroscopic approaches, especially Fourier-transform infrared (FTIR) or Raman spectroscopy, were subsequently employed to identify polymers and classify them by their characteristic absorbance wavelengths. The size, shape and sort of polymer of the discovered particulates were quantified. In recognition of their importance in causing profound lung buildup, particulates less than 10 µm required special consideration when categorizing them. Morphological classification included particulate and fibrous forms. Demographic details and clinical history were recorded for all respondents. During the bronchoscopy procedure, a standardized questionnaire was used to record respiratory signs, including wheezing, breathing difficulties with exertion and a persistent cough. Forced expiratory volume in one second (FEV_1_) readings have been monitored and displayed as percentages of anticipated readings following lung function examinations, in conformity with standard guidelines. For the purpose of determining inflammation-associated cell counts, comprising neutrophil and eosinophil proportions, BAL fluid was also subjected to cellular analysis. Available radiological findings were reviewed to assess parenchymal lung involvement.

## Ethical considerations:

The respondents were clearly informed of the nature of the research and provided informed consent. Every possible measure was taken to protect the patient's data. The institutional ethics committee granted ethical clearance to conduct the current survey.

## Statistical analysis:

The data were analysed using SPSS. The t-test and chi-square test were used to compare the micro-plastic-exposed and non-exposed groups.

## Results:

Among 92 participants, 69 (75%) were AMP-positive (detectable airborne micro-plastics in BAL fluid), while 23 (25%) were AMP-negative (Table 1 - see PDF). The current analysis revealed a mean age of 44.6 ± 11.2 (in years), with no statistically significant disparity (p = 0.48). Male predominance was noted (58.7%). Ninety-two respondents who underwent diagnostic bronchoscopy were included in the present analysis. A substantial proportion of those surveyed had particles of micro-plastic in their BAL fluid, implying that this particular group is frequently contaminated with plastic particles by breath. The concentration of micro-plastics fluctuated substantially among those with positive findings, ranging from comparatively small to very large particle burdens, suggesting considerable inter-individual disparities in exposure. The vast majority of the tiny particles had a diameter under 10 µm, comparable to sizes that could penetrate the distal regions of the respiratory tract. The most widely understood elements were polyethylene and polypropylene, in line with the polymer content analysis. Overall, the observed distribution closely mirrored patterns previously reported in environmental air samples and human lung tissue. Respondents displaying detectable tiny particles in their BAL fluid were more likely than those without them to report symptoms related to breathing, namely wheezing, breathing difficulties with exertion and a persistent cough. Additionally, a pulmonary health analysis found that people with exposure had lower mean FEV_1_ readings, suggesting a link between micro-plastics build up and impaired lung function.

## Discussion:

The present study contributes meaningful evidence to the evolving understanding of inhaled micro-plastics as an emerging respiratory exposure of clinical relevance. The revelation of particulates in BAL fluid in a considerable proportion of cases underscores the pervasiveness of environmental exposure and verifies that these particulates can reach the lower airways. The observed pattern supports growing fears that micro-plastics entering the airways might accumulate in distal lung areas, where biological responses are more likely to develop, rather than being confined to the surrounding tissues or upper airways. Our observations largely agree with previously published human studies reporting the presence of micro-plastics in respiratory samples. Pandey *et al.* demonstrated micro- plastic detection in BAL fluid in nearly two-thirds of individuals undergoing bronchoscopy, prevalence comparable to that observed in the present cohort [5]. In a comparable manner, Padarya *et al.* found micro-plastics in every BAL sample investigated, suggesting that inhalational exposure is prevalent even among individuals without identified occupational risk [6]. Extending these findings, Amato-Lourenço *et al.* identified micro-plastic particles embedded within resected lung tissue, providing compelling evidence that inhaled micro-plastics may persist beyond transient airway exposure and potentially accumulate within pulmonary parenchyma [7]. The insights of Padarya *et al.* who claimed that nearly all measured particulates were within a breathable size range that permitted them to penetrate deep into the airways, are closely paralleled by the present investigation's preponderance of particulates smaller than 10 µm [6]. Comparable size distributions have also been described by Jenner *et al.* in post-mortem lung samples, reinforcing the likelihood that smaller particles evade mucociliary clearance mechanisms [8]. In contrast, environmental air studies by Wright and Kelly have reported a higher proportion of larger micro-plastic particles, highlighting a clear distinction between environmental sampling and biological deposition patterns, likely reflecting selective retention within the respiratory tract [9]. In the context of polymers, the most frequently encountered polymers in the current research investigation were polyethylene and polypropylene. The pattern in question, which illustrates the widespread implementation of these elements in wrapping, textiles and household items, aligns with observations by Pandey *et al.* and Jenner *et al.* as well as numerous environmental research studies [5, 8]. Padarya *et al.* similarly identified polymers such as polyvinyl chloride and polystyrene, which were also detected in our cohort, though less frequently [6]. Morphologically, particulate micro-plastics were more common than fibrous forms in the present study. This observation is consistent with findings by Padarya *et al.* and Amato- Lourenço *et al.* both of whom reported a predominance of irregular fragments in BAL fluid and lung tissue, respectively [6, 7]. Talau *et al.* uncovered fibres as the dominant micro-plastic form in their analysis [3]. This apparent discrepancy may reflect differences in aerodynamic behaviour, deposition efficiency, or clearance dynamics, with particulate forms potentially more likely to reach and persist within distal airways. Furthermore, an increased likelihood of respiratory ailments, including continuous cough, wheezing and dyspnoea after vigorous activity, was associated with the micro-plastic verification in the present investigation. These outcomes correspond with the complaints described by Pandey *et al.* [5]. When comparing residents living in places with substantial amounts of airborne plastic particles. The observation of lower mean FEV_1_ values among exposed individuals further suggests early functional impairment. In contrast, Vianello *et al.* reported minimal functional impact in short-term exposure models, indicating that chronicity and cumulative exposure may play a decisive role in clinical manifestation [10]. Substantial biological validity can be inferred from the increase in inflammatory cell populations observed in BAL fluid among affected people. In line with scientific studies demonstrating that tiny plastic particles spur oxidative stress and cytokine activation, heightened proportions of neutrophils and eosinophils imply protracted airway irritation [11, 12]. Studies hence emphasize the necessity of incorporating micro-plastic monitoring into surveillance of environmental and occupational exposure [13, 14]. Despite these strengths, certain limitations merit consideration. It is impossible to draw precise inferences about the time correlations between exposure and breathing effects due to the cross-sectional design. Disparities may have influenced the recognition of tiny plastic particles in the method of analysis and discovery specificity, which might have accounted for disparities in the rate of identification between analyses. Furthermore, exposure diversity might have been contributed to by overlooked factors such as lifestyle and environment. Despite these shortcomings, direct BAL analysis, comprehensive particle analysis and the integration of clinical, functional and inflammatory findings benefit the currently ongoing study. To clarify dose-response associations and ongoing clinical consequences of chronic micro-plastic inhalation, longitudinal analyses using standardized screening methods are required.

## Conclusion:

Data shows that airborne micro-plastics are commonly present in the lower respiratory tract of urban adults with respiratory symptoms and are associated with airway inflammation, impaired lung function and increased symptom burden. Thus, those airborne micro-plastics may represent an emerging environmental risk factor for chronic respiratory disease and warrant further investigation and preventive public health strategies.

## References

[1] Jahedi F (2025). BMC Cardiovasc Disord..

[2] Rybalchenko Y (2024). The Medical and Ecological Problems..

[3] Talau SA (2025). Diagnostics (Basel)..

[4] Qiu L (2023). Environ Sci Technol..

[5] Pandey M (2025). J Pharm Bioallied Sci..

[6] Padarya SK (2025). J Pharm Bioallied Sci..

[7] Amato-Lourenço LF (2021). J Hazard Mater..

[8] Jenner LC (2022). Sci Total Environ..

[9] Wright SL, Kelly FJ (2017). Environ Sci Technol..

[10] Vianello A (2019). Sci Rep..

[11] Holz O (2022). Sci Rep..

[12] Wang P (2025). Ital J Pediatr..

[13] Zakynthinos GE (2025). Cureus..

[14] Tokito T (2025). Respirology..

